# E-Sports Competition Analysis Based on Intelligent Analysis System

**DOI:** 10.1155/2022/4855550

**Published:** 2022-07-08

**Authors:** Yao Lu, Hao Chen, Hongqiao Yan

**Affiliations:** School of Sport Communication and Information Technology, Shandong Sport University, Jinan 250000, China

## Abstract

To improve the analysis effect of e-sports competitions, this paper studies the intelligent analysis methods of e-sports, compares the advantages and disadvantages of various multi-classification methods, and innovatively designs a two-layer SVM classifier structure for four types of motor imagery EEG signals. Moreover, this paper uses the competition data set to test the classification accuracy of the designed double-layer SVM classifier structure and compares it with the DAG-SVM multi-classification method. The research results show that the e-sports competition analysis system based on the intelligent analysis system proposed in this paper has a good e-sports competition analysis effect, and has a good effect in e-sports competition prediction.

## 1. Introduction

With the rapid development of e-sports, the relevant e-sports talents are relatively scarce, and the lack of talents has also become a bottleneck in the development of e-sports. As the main places where talents gather, the development of e-sports in colleges and universities will help all sectors of society pay attention to healthy e-sports sports and promote its rational development. Moreover, it also helps to cultivate more esports talents and push the esports industry in the right direction.

E-sports usually means using electronic equipment as a carrier to participate in games developed by various game developers, compete with each other, and achieve projects similar to traditional competitive competitions such as Go and badminton [1]. In this way, e-sports refers to the use of various electronic devices, such as mobile phones, iPads, PCs, etc., as sports equipment, to carry out mental, physical and other confrontation sports between players [2]. In this sport, if it is a team event, it can greatly cultivate team spirit, or if it is an individual event, it can continuously improve the athletes' various willpower and coordination and control capabilities [3].

Due to the youthfulness of e-sports itself and the roughness of the past, not many scholars have devoted their academic energy to it in the past, and the traditional Tour de France and other technologies such as game replay have no obvious effect on e-sports., so this area has not been studied by relevant scholars until recent years, and the general research methods used are still some primary research methods [4]. Some machine learning methods used in literature [5], whether it is a traditional machine learning algorithm such as k-nearest neighbor (KNN), or a currently popular neural network algorithm or even a deep neural network algorithm for deep learning, etc. used in related research analysis. So for the entire traditional machine learning algorithms, such as KNN, SVM, logistic regression, etc., are some algorithms that have been studied since the last century and have been developed so far, so I will not go into details here, but if you consider the If we think about the whole game winning and losing analysis model from a different angle, and convert it into a small dictionary, a special kind of natural language processing model (NLP) in the form of reassociation, we will find that some of today's natural language processing methods have an impact on it. Adaptability [6]: the natural language recognition model was also proposed in the last century and has been continuously studied so far. Most of the models are modeled by using expert rules of grammatical features such as syntax and lexical features. Later, due to the rapid increase in the magnitude of the data to be processed, at the beginning of this century, people mostly adopted It is a supervised feature engineering method built on the above features to model [7]. For such large-scale data, a series of problems such as how to minimize the label training brought by supervision without using feature engineering are put in front of us [8]. In the subsequent development, due to the continuous application and addition of neural network technology with the new method of deep learning, it has played a great role in various fields. Especially in tasks such as speech analysis and image processing and recognition, which are traditionally difficult to deal with by machine learning, after using deep neural networks, the improvement is obvious, and each performance index exceeds traditional solutions. The provided scheme [9]. However, due to the particularity of the textual information itself, even for simple synonyms such as “Sunday” and “Sunday”, the difference in their performance is huge, unlike in the fields of speech analysis and image recognition, the signal data are similar, which means that we need more background information to deal with these problems [10].

Literature [11] believes that there is a difference between the broad and narrow senses of e-sports: in a broad sense, e-sports is the implementation and development of traditional sports through e-sports; in a narrow sense, both the connotation and extension of e-sports should be Clearly, we can define e-sports. However, whether in a broad or narrow sense, it is necessary to determine that e-sports itself is a sport, and then we must look at problems from a developmental perspective, and we must also use a developmental perspective to look at the impact of e-sports on social development. Influence [12]: Ivanytska [13] believes that e-sports refers to sports that players perform by operating computers. That is, players need to operate a computer and rely on the software and hardware facilities of the computer to perform sports. And e-sports should have a unified set of rules to implement like sports in real life, but the environment in real life is moved to the competitive competition in virtual environment. E-sports has two basic attributes: e-sports and sports. “Electronics” refers to the core of science and technology and the support of computer hardware and software facilities, which is the equipment and venues required by traditional sports in real life [14]. And “sports” is the essence of all sports. No matter how e-sports is introduced, its core must still be confrontation and competition [15]. Literature [16] made four definitions for the concept of e-sports: first, e-sports must be carried out under the constraints of rules, which first emphasizes the competition and confrontation between people; second, the software and hardware facilities of e-sports are Like the various equipment venues of traditional sports, it is the basic carrier for carrying e-sports; third, e-sports can also promote exchanges between people; fourth, participating in e-sports can cultivate and develop participants' various skills. This ability also greatly promotes the healthy growth of its physical and mental health.

## 2. Intelligent e-Sports Competition Analysis Algorithm

### 2.1. EEG Signal Processing Algorithm

When people are thinking about e-sports in a certain part of their body such as their hands and feet, or in their minds, the activation energy of a certain part of the cerebral cortex that controls the e-sports behavior of the body increases. This state is called Event-related Synchronization (ERS). However, the activation energy of the part of the cortex opposite this part of the cerebral cortex is reduced, and this state is called Event-related De-synchronization (ERD).

Some research groups have used ERS/ERD, which imagines the e-sports behavior of a certain part of the body, in BCI. When we imagine gaming behaviors in a certain part of the body, there will be ERS/ERDs that activate energy states in the relevant part of the cerebral cortex that controls the gaming behaviors of the body. For example, when we imagine hand e-sports behavior, the activation energy of *μ* and *β* in the same lateral cortical part that controls hand e-sports behavior is enhanced, which is the ERS state. The activation energies of *μ* and *β* in the opposite lateral cortical parts that control hand esports behavior are reduced, which is the ERD state.

### 2.2. Preprocessing Algorithms

EEG signals are very weak and prone to interference. To filter out noise and artifacts and improve the signal-to-noise ratio and spatial resolution, EEG signals must be preprocessed before feature extraction and pattern classification. At present, the preprocessing methods of EEG signal mainly include spatial filter and frequency domain filter.

Common average reference method (CAR) for EEG signal preprocessing. First, the average of all the collected channel data is calculated, and then the calculated average is subtracted from each channel data, and finally, the new data after CAR filtering are obtained. The calculation formula of the CAR algorithm is as follows:

(1) $$\begin{array}{c} V_{i}^{CAR}=V_{i}^{ER}-\frac{1}{n}\sum_{j=1}^{n}V_{j}^{ER}. \end{array}$$

In the formula, *V*_*i*_^*CAR*^ is the signal of the *i*th channel after CAR filtering, and *V*_*i*_^*ER*^ is the signal of the *i*th channel minus the reference electrode signal.

The Laplacian algorithm processes EEG signals. First, the average number of several channels of data near each channel of data is selected, and then the calculated average number is subtracted from the data of this channel, and the new data after Laplacian filtering are obtained after processing the data of all channels. The Laplacian formula is as follows:

(2) $$\begin{array}{c} V_{i}^{LAP}=V_{i}^{ER}-\sum_{j\in S_{i}}g_{ij}V_{j}^{ER}. \end{array}$$

Among them, *g*_*ij*_=1/*d*_*ij*_/∑_*j*∈*S*_*i*__1/*d*_*ij*_, *V*_*i*_^*LAP*^ is the Laplacian filtered signal of the original signal of the ith channel, *V*_*i*_^*ER*^ is the potential difference between the electrode of the ith channel and the reference electrode, and *S*_*i*_ is the electrodes around the ith channel. Meanwhile, *d*_*ij*_ is the scalp surface distance between the *i*-th and *j*-th channels.

In the actual calculation of Laplacian, each channel selects the adjacent 4 channels to calculate the average, and then the data of each channel is obtained by subtracting the corresponding average filtered data. The mathematical formula is as follows:

(3) $$\begin{array}{c} V_{i}^{LAP}=V_{i}^{ER}-\frac{1}{4}\sum_{j\in S_{i}}V_{j}^{ER}. \end{array}$$

Among them, *S*_*i*_ is the number of the four electrodes around the desired electrode.

Frequency domain filtering methods include frequency domain bandpass filtering and notch filtering. The frequency domain filtering of EEG signal can filter out the power frequency signal of 50 Hz and reserve the specific frequency band (8 − 26 Hz) to extract the EEG signal related to motor imagery and improve the signal-to-noise ratio of the signal. Filters are mainly divided into IIR filters and FIR filters. The system function of the filter is:

(4) $$\begin{array}{c} H\left(z\right)=\frac{A\left(z\right)}{B\left(z\right)}=\frac{a_{0}+a_{1}z^{-1}+a_{2}z^{-2}+\ldots +a_{N}z^{-N}}{1+b_{1}z^{-1}+b_{2}z^{-2}+\ldots +b_{M}z^{-M}}. \end{array}$$

In the formula, when there is *M* ≥ 1, the impulse response of the system is infinitely long, which is called IIR filter. When there is *B*(*z*)=1, the response length of the system is *N*+1, which is called FIR filter.

### 2.3. Feature Extraction Algorithm

Feature extraction refers to extracting the information in the EEG signal that can best express human thinking tasks. The most different information between different thinking tasks can be extracted, and the classification accuracy can be significantly improved after training the classifier with such characteristic information. At present, the commonly used EEG signal feature extraction algorithms include time domain analysis method, frequency domain analysis method, time-frequency domain analysis method, and common spatial pattern.

The power spectrum estimation of EEG signal is a frequency domain analysis method. It transforms the EEG signal in the time domain into the energy change of the EEG signal in the frequency domain with frequency. If the sampling value *x*_*N*_(*n*) of the N points of the EEG signal *X*(*n*) is set, and *X*_*N*_(*e*^*jω*^) is obtained after Fourier transform, the power spectrum *P*(*e*^*jω*^) of *X*(*n*) is estimated as:

(5) $$\begin{array}{c} \hat{P}\left(e^{j\omega }\right)=\frac{1}{N}{\left|X_{N}\left(\omega \right)\right|}^{2}. \end{array}$$

The main idea of the short-time Fourier transform is to use a sliding window function W(t) to analyze the signal X(t) , and then perform Fourier transform on the windowed signal:

(6) $$\begin{array}{c} \mathrm{STF}T_{x}\left(t,\Omega \right)=\int_{-\infty }^{+\infty }x\left(\tau \right)w\left(\tau -t\right)e^{-j\Omega \tau }d\tau . \end{array}$$

In the formula, Ω is the angular frequency, and the window function W(t) generally chooses the Hanning window.

When the short-time Fourier transform is used to analyze the signal, the determination of the window function is very critical. We adjust the size of the window to adjust the resolution in time and frequency. We use a certain window to select the signal for Fourier transform, and then slide the window to select the next signal for the same analysis. Finally, the time-spectrogram will be obtained, and the power spectral density is:

(7) $$\begin{array}{c} P\left(t,\Omega \right)=\frac{{\left|\mathrm{STF}T_{x}\left(\Omega ,t\right)\right|}^{2}}{2\pi }. \end{array}$$

In the process of analyzing the signal, the local analysis ability of the wavelet transform is very good, that is, the resolution of time and frequency is high. The main idea is to use the window function that can change at any time to analyze the detailed information of each scale of the signal. There is a higher frequency resolution in the low-frequency band, and a higher time resolution in the high-frequency band.

If function *ψ*(*t*) ∈ *L*^2^(*R*) satisfies:

(8) $$\begin{array}{c} \int_{R}\psi \left(t\right)dt=0. \end{array}$$

Then, *ψ*(*t*) is called a basic wavelet.

The above wavelet function is transformed by scaling and translation, that is, by scaling factor a and translation factor *b*, the transformed wavelet function is:

(9) $$\begin{array}{c} \psi_{a,b}\left(t\right)={\left|a\right|}^{-1/2}\psi \left(\frac{t-b}{a}\right). \end{array}$$

In the formula, the scale factor a determines the frequency domain information in the wavelet transform, and the translation factor *b* determines the time domain information. The continuous wavelet transform of any function *f*(*t*) ∈ *L*^2^(*R*) is as follows:

(10) $$\begin{array}{c} WT_{f}\left(a,b\right)=\langle f\left(t\right),\psi_{a,b}\left(t\right)\rangle ={\left|a\right|}^{-1/2}\int_{-\infty }^{+\infty }f\left(t\right)\psi \left(\frac{t-b}{a}\right)\text{d}t. \end{array}$$

If discrete wavelet transform is required, *a* and *b* need to be discretized. For example, if we set the scale factor *a*=2^*j*^ and the translation factor *b*=2^*j*^*k*, the discrete wavelet transform is:

(11) $$\begin{array}{c} WT_{f}\left(a,b\right)=2^{-j/2}\int_{R}f\left(t\right)\psi \left(2^{-j}t-k\right)\text{d}t. \end{array}$$

Mallat algorithm is a fast algorithm for wavelet decomposition and reconstruction proposed by Mallat in 1989. According to the Mallat algorithm, the signal *f*(*t*) can be decomposed to obtain:

(12) $$\begin{array}{c} f\left(t\right)=A_{L}+\sum_{j=1}^{L}D_{j}=A_{L}+D_{L}+D_{L-1}+\ldots +D_{1}. \end{array}$$

In the formula, *L* is the number of decomposition layers, *A*_*L*_ is the approximate component of the *L*th layer, and *D*_*j*_ is the detail component at the *j*-th scale. We set the sampling frequency of the signal *f*(*t*) as *f*_*s*_, and the frequency of the signal can be divided into L+1 subbands, then the subbands corresponding to each component *A*_*L*_, *D*_*L*_, *D*_*L*−1_, ⋯, *D*_1_ are:

(13) $$\begin{array}{c} \left[0,\frac{f_{s}}{2^{L+1}}\right],\left[\frac{f_{s}}{2^{L+1}},\frac{f_{s}}{2^{L}}\right],\left[\frac{f_{s}}{2^{L}},\frac{f_{s}}{2^{L-1}}\right],\ldots ,\left[\frac{f_{s}}{2^{2}},\frac{f_{s}}{2}\right]. \end{array}$$

**Figure 1:**
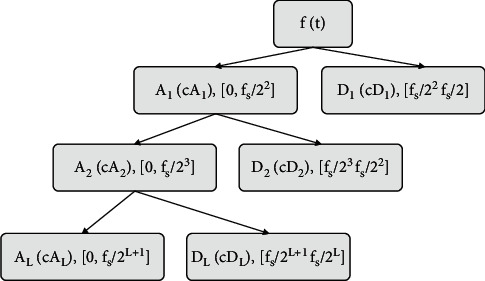
L-layer decomposition of signal *f*(*t*).

The corresponding wavelet coefficients of each layer are *cA*_*L*_, *cD*_*L*_, *cD*_*L*−1_,…, *cD*_1_ in turn. The Mallat tower decomposition is shown in Figure 1.

The EEG signal is formed by the superposition of many source signals, and CSP analyzes it according to the method of decomposing the source signals. We assume that the EEG signals collected when imagining event A and event B are *X*_*A*_ and *X*_*B*_, *X*_*A*_ B and *X*_*B*_ are represented as:

(14) $$\begin{array}{c} X_{A}=\left[C_{A}C_{C}\right]\left[\begin{array}{c} S_{A}\\ S_{C} \end{array}\right],\\ X_{B}=\left[C_{B}C_{C}\right]\left[\begin{array}{c} S_{B}\\ S_{C} \end{array}\right]. \end{array}$$

In the formula, *S*_*A*_ is the source signal caused by event A, *S*_*B*_ is the source signal caused by event *B*, and *S*_*C*_ is the source signal caused by event A and B together. Meanwhile, *C*_*A*_, *C*_*B*_, *C*_*C*_ are the spatial patterns of the three types of source signals, respectively, and the idea of CSP is to estimate *C*_*A*_*S*_*A*_ and *C*_*B*_*S*_*B*_ related to events *A* and *B*. If the spatial filters of events *A* and *B* obtained by CSP are *F*_*A*_ and *F*_*B*_, for the input *X*, there is the following relationship:

(15) $$\begin{array}{c} C_{A}S_{A}=F_{A}XC_{B}S_{B}=F_{B}X. \end{array}$$

When event *A* is imagined, *C*_*A*_*S*_*A*_ relative to event *A* is greater than when event *B* is imagined. Meanwhile, when event B is imagined, *C*_*B*_*S*_*B*_ relative to event *B* is greater than when event A is imagined. According to this principle, different imaginary events can be classified, so CSP converts the events to extract the source signals *S*_*A*_ and *S*_*B*_ related to the events.

The procedure of the CSP algorithm will now be described. We set *X*_*A*_ and *X*_*B*_ to be the EEG signals when events A and B are imagined, then the normalized covariance matrices of *X*_*A*_ and *X*_*B*_ are:

(16) $$\begin{array}{c} R_{A}=\frac{X_{A}X_{A}^{T}}{\mathrm{trace}\left(X_{A}X_{A}^{T}\right)}\\ R_{B}=\frac{X_{B}X_{B}^{T}}{\mathrm{trace}\left(X_{B}X_{B}^{T}\right)}. \end{array}$$

In the formula, *X*_*A*_^*T*^ is the transpose of *X*_*A*_ and trace(*X*_*A*_*X*_*A*_^*T*^) is the trace of *X*_*A*_*X*_*A*_^*T*^. The *R*_*A*_ of multiple groups of *X*_*A*_ is averaged to obtain $\bar{R_{A}}$, and the average of *R*_*B*_ of multiple groups of *X*_*B*_ is also averaged to obtain $\bar{R_{B}}$, and the mixed covariance matrix *R* is:

(17) $$\begin{array}{c} R=\bar{R_{A}}+\bar{R_{B}}. \end{array}$$

After the mixed covariance matrix *R* eigenvalues are decomposed, we get

(18) $$\begin{array}{c} R=U\Sigma U^{T}. \end{array}$$

In the formula, *U* is the eigenvector of the *R* matrix, and Σ is the eigenvalue matrix corresponding to the eigenvector of *R*. The whitening matrix *P* is constructed:

(19) $$\begin{array}{c} P=\Sigma^{-1/2}U^{T}. \end{array}$$

Then, $\bar{R_{A}}$ and $\bar{R_{B}}$ are whitened and transformed into:

(20) $$\begin{array}{c} S_{A}=P\bar{R_{A}}P^{T}S_{B}=P\bar{R_{B}}P^{T}. \end{array}$$

*S*_*A*_ and *S*_*B*_ are decomposed into eigenvalues to get

(21) $$\begin{array}{c} S_{A}=U_{A}\Sigma_{A}U_{A}^{T}S_{B}=U_{B}\Sigma_{B}U_{B}^{T}. \end{array}$$

It is easy to know that *S*_*A*_ and *S*_*B*_ have the following characteristics:

(22) $$\begin{array}{c} U_{A}=U_{B}=U\Sigma_{A}+\Sigma_{B}=I. \end{array}$$

This shows that the sum of the elements corresponding to the diagonals of Σ_*A*_ and Σ_*B*_ is 1, the maximum value in Σ_*A*_ of *S*_*A*_ corresponds to the minimum value in Σ_*B*_ of *S*_*B*_, and the maximum value in Σ_*B*_ of *S*_*B*_ corresponds to the minimum value in Σ_*A*_ of *S*_*A*_. If the Σ_*A*_ value of *S*_*A*_ is sorted from large to small, and U is arranged accordingly, so that the Σ_*B*_ value of *S*_*B*_ is sorted from small to large. Therefore, it can be classified according to the difference between the first *m* and the last *m* values in the sorted Σ_*A*_ and Σ_*B*_. Accordingly, the spatial filter matrix is designed:

(23) $$\begin{array}{c} SF=U^{T}P. \end{array}$$

According to the sorted Σ_*A*_ and Σ_*B*_, *U*, they are also sorted accordingly, and then the first *m* rows and the last *m* rows of U are selected to form a new *U*′, and the spatial filter matrix is obtained:

(24) $$\begin{array}{c} SF^{\prime }=U^{\prime T}P. \end{array}$$

For the EEG signal *X*_*k*_, *Z*_*k*_=*SF*′*X*_*k*_ is used to obtain the projected EEG signal *Z*_*k*_. According to the *m* value, the line *Z*_*p*_ is taken out from the projected EEG signal *Z*_*k*_ to calculate the variance, and then the eigenvalues are obtained by the following calculation:

(25) $$\begin{array}{c} f_{p}=\text{lg}\left(\frac{\mathrm{var}\left(Z_{p}\right)}{\sum_{i=1}^{2m}\mathrm{var}\left(Z_{i}\right)}\right). \end{array}$$

In the formula, var() represents the variance, and there is *p*=1,2,…, 2*m*. After processing by CSP algorithm, 2m-dimensional feature vector can be obtained for classification.

### 2.4. Support Vector Machines

The Support Vector Machine (SVM) classification method is very effective in pattern classification problems of small samples, nonlinear and high-dimensional data sets, and it can be applied to machine learning problems such as function fitting.

**Figure 2:**
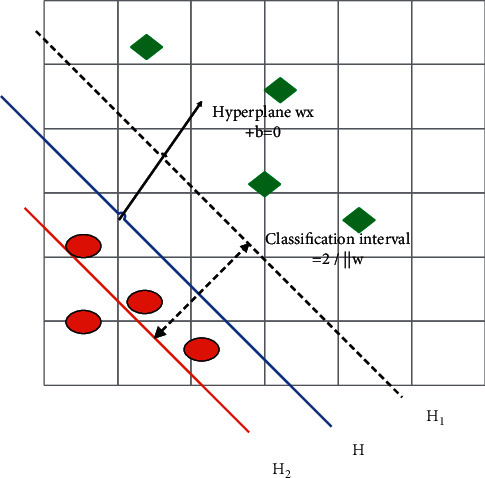
Schematic diagram of linear time-divisible SVM classification.

When the sample data are linearly separable, the main goal of SVM is to find the classification hyperplane that can accurately separate the two types of sample data and has the largest classification interval in the sample space. As shown in Figure 2, the red hollow circles and green small triangles in the figure represent the sample data of two different motor imagery tasks, respectively. H is the classification hyperplane of the two types of sample data, *H*_1_ and *H*_2_ are the classification boundaries made by the data points closest to H in the two types of sample data, and the distance between *H*_1_ and *H*_2_ is called the classification interval.

We set the sample as (*x*_*i*_, *y*_*i*_), *x*_*i*_ is the *i*th input sample, *y*_*i*_ is the class label of the input sample *x*_*i*_, there is *y*_*i*_ ∈ {−1, +1}. *i*=1,2,…, *l*, *l* is the number of input samples. The discriminant function is: *f*(*x*)=*w* · *x*+*b*, *w* is the weight vector, *b* is the classification threshold, then the classification hyperplane is *w* · *x*+*b* ≤ 0, so that we have:

(26) $$\begin{array}{c} w\cdot x_{i}+b\geq 1,y_{i}=1,\\ w\cdot x_{i}+b\leq 1,y_{i}=-1. \end{array}$$

That is, it merges into:

(27) $$\begin{array}{c} y_{i}\left(w\cdot x_{i}+b\right)\geq 1. \end{array}$$

After *f*(*X*) is normalized, *x* satisfies |*f*(*x*)| ≥ 1, and the nearest *x* to H satisfies |*f*(*x*)|=1, then the distance from *x* to H is:

(28) $$\begin{array}{c} d=\frac{\left|f\left(x\right)\right|}{\left\|w\right\|}=\frac{1}{\left\|w\right\|}. \end{array}$$

Therefore, the classification interval of the two types of sample data is 2/‖*w*‖. If we want to maximize the classification interval, we want to minimize ‖*w*‖. This can be transformed into a quadratic programming problem:

(29) $$\begin{array}{c} \min_{w,b}\frac{1}{2}{\left\|w\right\|}^{2}. \end{array}$$

*S*.*t*. *y*_*i*_(*x* · *w*+*b*) ≥ 1

The constructed Lagrangian function is:

(30) $$\begin{array}{c} L\left(w,b,\alpha \right)=\frac{1}{2}{\left\|w\right\|}^{2}-\sum_{i=1}^{l}\alpha_{i}\left[y_{i}\left(w\cdot x_{i}+b\right)-1\right]. \end{array}$$

In the formula, the *α*_*i*_ parameter is called the Lagrange multiplier. The Lagrangian function takes the partial derivatives of the *w* and *b* parameters respectively, and then the optimization problem can be transformed into a dual problem to solve:

(31) $$\begin{array}{c} \text{max}\sum_{i=1}^{l}\alpha_{i}-\frac{1}{2}\sum_{i=1}^{l}\sum_{j=1}^{l}y_{i}y_{j}\alpha_{i}\alpha_{j}\left(x_{i},x_{j}\right)\\ s.t.\sum_{i=1}^{l}y_{i}\alpha_{i}\leq 0,\alpha_{i}\geq 0. \end{array}$$

According to this, *α*^*∗*^ is obtained as the optimal solution, and we then obtain:

(32) $$\begin{array}{c} w^{*}=\sum_{i=1}^{l}y_{i}\alpha_{i}^{*}x_{i},b^{*}\\ =y_{j}-\sum_{i=1}^{l}y_{i}\alpha_{i}\left(x_{i},x_{j}\right). \end{array}$$

Parameters *α*^*∗*^, *w*^*∗*^ and *b*^*∗*^ satisfy *α*_*i*_^*∗*^[*y*_*i*_(*w*^*∗*^ · *x*_*i*_+*b*^*∗*^) − 1]=0, *i*=1,2,…, *l*. This formula shows that the interval is 1 x corresponds to *α*^*∗*^ ≠ 0, and other *x* corresponds to *α*^*∗*^=0. Therefore, *w* consists of *l*_*s*_ ≤ *l* eigenvectors, and *w* is:

(33) $$\begin{array}{c} w=\sum_{i=1}^{l_{s}}y_{i}\alpha_{i}x_{i},\alpha^{*}\neq 0. \end{array}$$

Finally, the available classification function is:

(34) $$\begin{array}{c} g\left(x\right)=\text{sgn}\left\{\left(w^{*}\cdot x\right)+b^{*}\right\}\\ =\text{sgn}\left\{\sum_{i=1}^{l}\alpha_{i}^{*}y_{i}\left(x_{i},x\right)+b^{*}\right\}. \end{array}$$

In the formula, sgn(·) is the sign function.

When the sample data are linearly inseparable, the loose variable *ξ*_*i*_ is introduced, and the constraints are transformed into:

(35) $$\begin{array}{c} y_{i}\left(w\cdot x_{i}+b\right)\geq 1-\xi_{i},\left(\xi_{i}\geq 0,i=1,2,\ldots ,l\right). \end{array}$$

The optimal problem is transformed into:

(36) $$\begin{array}{c} \min\frac{1}{2}{\left\|w\right\|}^{2}+C\sum_{i=1}^{l}\xi_{i}\\ s.t.y_{i}\left(w\cdot x_{i}+b\right)\geq 1-\xi_{i}. \end{array}$$

In the formula, *C* is called the penalty parameter that controls the number of misclassified samples.

The constructed Lagrangian function is:

(37) $$\begin{array}{c} L\left(w,b,\xi ,\alpha \right)=\frac{1}{2}{\left\|w\right\|}^{2}-\sum_{i=1}^{l}\alpha_{i}\left[y_{i}\left(w\cdot x_{i}+b\right)-1+\xi_{i}\right]+\frac{C}{2}\sum_{i=1}^{l}\xi_{i}. \end{array}$$

The Lagrangian function is solved and transformed into the solution dual problem:

(38) $$\begin{array}{c} \text{max}\sum_{i=1}^{l}\alpha_{i}-\frac{1}{2}\sum_{i=1}^{l}\sum_{j=1}^{l}y_{i}y_{j}\alpha_{i}\alpha_{j}\left(x_{i},x_{j}\right)\\ s.t.\sum_{i=1}^{l}y_{i}\alpha_{i}=0,0\leq \alpha_{i}\leq C. \end{array}$$

Nonlinear sample data are classified by using a nonlinear SVM. The most important method of SVM classification is to use the kernel function to map the sample data to a new high-dimensional feature space F, and use linear SVM for classification in *F*. We set *ϕ*(*x*) as the nonlinear mapping from the input sample data space *X* to the new feature space *F*, then the classification hyperplane transformation in the nonlinear case is:

(39) $$\begin{array}{c} w\cdot \phi \left(x\right)+b=0. \end{array}$$

The decision function is:

(40) $$\begin{array}{c} f\left(x\right)=\text{sgn}\left[w\cdot \phi \left(x\right)+b\right]. \end{array}$$

Since it involves inner product operations in SVM, such as 〈*x*_*i*_, *x*_*j*_〉 and 〈*ϕ*(*x*_*i*_), *ϕ*(*x*_*j*_)〉, the concept of kernel function is introduced. We set *K* as a kernel function, then for all *x*_*i*_, *x*_*j*_ ∈ *X*, *K*(*x*_*i*_, *x*_*j*_)=〈*ϕ*(*x*_*i*_) · *ϕ*(*x*_*j*_)〉 is satisfied.

The kernel functions commonly used in SVM classification are as follows:

  Linear kernel function: *K*(*x*_*i*_, *x*_*j*_)=*x*_*i*_^*T*^*x*_*j*_
  Polynomial kernel function: *K*(*x*_*i*_, *x*_*j*_)=(*x*_*i*_^*T*^*x*_*j*_+1)^*q*^, *q* > 0
  Radial basis kernel function: *K*(*x*_*i*_, *x*_*j*_)=exp(−‖*x*_*i*_ − *x*_*j*_‖^2^/*σ*^2^)
  Sigmoid kernel function: *K*(*x*_*i*_, *x*_*j*_)=tanh(*βx*_*i*_^*T*^*x*_*j*_+*γ*)

### 2.5. Selection of SVM Kernel Function and Parameters

The SVM kernel function mainly maps the input sample data into a high-dimensional feature space. When different SVM kernel functions are selected, different classification hyperplanes will be generated, and when several kernel functions are used for the same sample, it will be found that the classification effects are different. Therefore, it is critical to use a better SVM kernel function. Among them, the radial basis kernel function (RBF) has a wide range of applications in the feature classification of EEG signals, regardless of the sample size and dimension, the RBF kernel function is applicable. Because the RBF kernel function has the advantages of simple calculation and fast training speed, this paper chooses the SVM classifier with RBF as the kernel function to classify the EEG signals.

If using RBF as the kernel function for classification, two parameters need to be optimized, namely the penalty parameter C and the RBF kernel parameter. When looking for the optimal (C, g) parameter pair, a combination of cross-validation (K-fold cross-validation, K-CV) and grid search is generally used. First, the parameters C and g take values within a certain range. When the parameter pairs (C, g) is selected, the K-CV method is used on the training data set to obtain the classification accuracy of the training data set. After that, the next set of parameter pairs (C, g) are selected for testing, and the test will not end until all parameter pairs (C, g) have been determined. Finally, the group (C, g) with the highest classification accuracy of the training data set is selected as the optimal parameter. If the highest classification accuracy rate in the training data set corresponds to multiple parameter pairs (C, g), the group (C, g) with the smallest C parameter is selected. If the selected minimum C parameter has multiple *g* parameters, the first group (C, g) is selected as the optimal parameter. However, if the selected C parameter is too large, it is easy to generate an over-learning state. Therefore, among the multiple parameter pairs (C, g) corresponding to the highest classification accuracy rate, the group with the smaller penalty parameter C should be selected as the optimal parameter.

**Figure 3:**
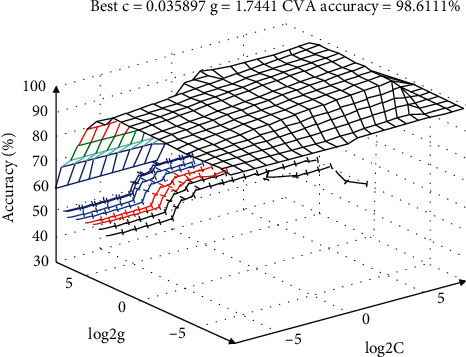
Result diagram of optimal parameter selection (3D view).

**Figure 4:**
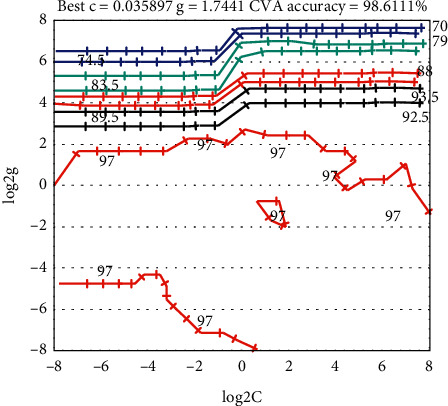
Result diagram of optimal parameter selection (contour map).

This paper uses the grid parameter optimization function SVMcgForClass in the libsvm-mat-2.89-3 [FarutoUltimate3.0] toolbox. The results of selecting the optimal SVM parameter pair (C, g) using the K-CV method and grid search method are shown in Figures 3 and 4, where *K* = 5. It can be seen from the figure that the optimal SVM parameter pair selected by the K-CV method and the grid search method for the data of the subjects is *C* = 0.035856 and *g* = 1.75326.

SVM is a two-class classifier. When classifying multi-class data, there are mainly the following two classification methods. 1. It expands the original two-class optimization problem so that it can find all multi-class classification functions at the same time, and complete multi-class classification at one time in the classification process.

The original question can be rewritten as:

(41) $$\begin{array}{c} \min\frac{1}{2}\sum_{m=1}^{k}{\left\|w_{m}\right\|}^{2}+C\sum_{i=1}^{l}\sum_{m\neq y_{i}}\xi_{i}^{m}s.t.\left(w_{i}\cdot x_{i}\right)+b_{i}\geq \left(w_{m}\cdot x_{i}\right)+b_{m}+2-\xi_{i}^{m},\xi_{i}^{m}\geq 0. \end{array}$$

In the formula, *i*=1,2, ⋯, *l*, *l* is the number of samples, and *m*=1,2,…*k*, *k* is the number of categories. The decision function *f*(*x*)=max_*i*_[(*w*_*i*_ · *x*)+*b*_*i*_] can be obtained, and the discriminant result is the *i*th class.

This paper uses a combination of multiple two-class SVM classifiers to design a multi-class classification method, mainly including:

#### 2.5.1. OVR-SVM

**Figure 5:**
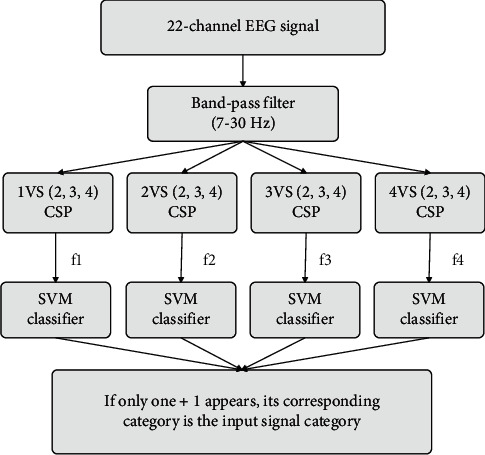
The structure of the OVR-SVM classifier.

When CSP extracts the EEG signal features of four types of motor imagery, one of the features is One-Versus-Rest CSP (OVR-CSP). After extracting four types of EEG signal features by this method, four SVMs are used to classify them respectively. If only one +1 appears in the classification results of the four SVM classifiers, the class corresponding to the classifier is the input signal class. This multi-class classifier structure designed above is called OVR-SVM, as shown in Figure 5.

#### 2.5.2. OVO-SVM

**Figure 6:**
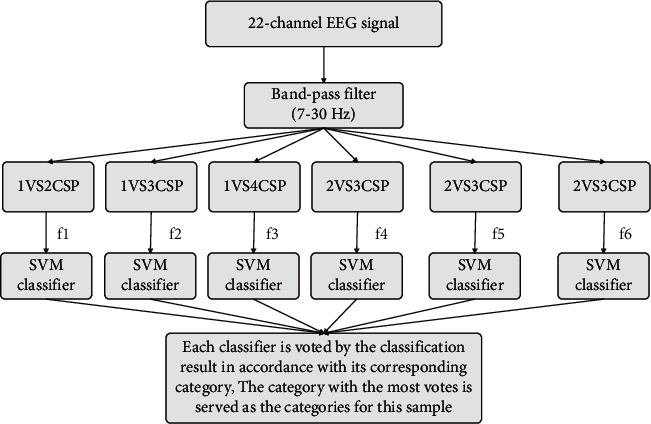
The structure of the OVO-SVM classifier.

Another method for feature extraction of four types of motor imagery EEG signals using the CSP algorithm is the One-Versus-One CSP (OVO-CSP) method. After extracting 4 types of EEG signal features in this way, 6 SVM classifications are used, and then the number of votes is counted to determine the final recognition output. The above classification method is called OVO-SVM, as shown in Figure 6.

#### 2.5.3. DAY-SVM

**Figure 7:**
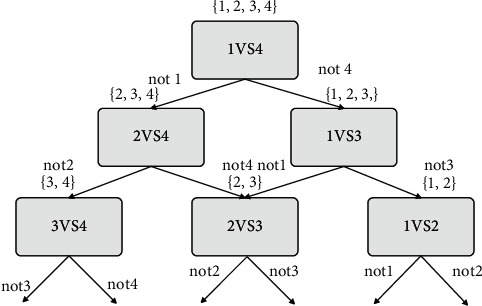
DAG-SVM schematic diagram.

DAG-SVM is a multi-class classifier structure designed based on the directed acyclic graph DAG proposed by Platt, mainly to solve the problem of misclassification and rejection of the OVO-SVM classifier structure. DAG-SVM is simple and easy to implement, and the test speed is fast. The principle of DAG-SVM for 4 categories is shown in Figure 7. However, the DAG-SVM classifier structure has the defect of error accumulation. If one of the classifiers is wrongly classified, it will affect the subsequent classification and make the final classification result wrong.

## 3. E-Sports Competition Analysis Based on Intelligent Analysis System

**Figure 8:**
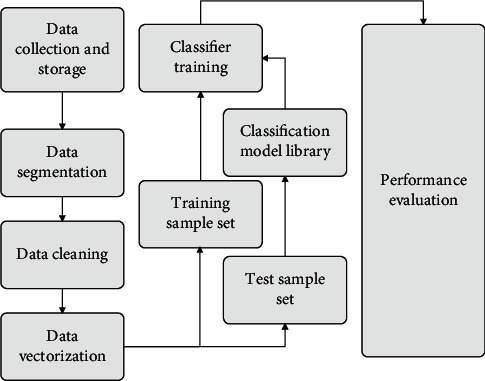
E-sports competition analysis system.

To better determine which classifier to use to build the final model, the modules operate independently and work in a variety of ways, so the system is more flexible.  Figure 8 shows the detailed modules of each module of the entire e-sports competition analysis system working with each other, and attaches the relevant flow chart.

**Figure 9:**
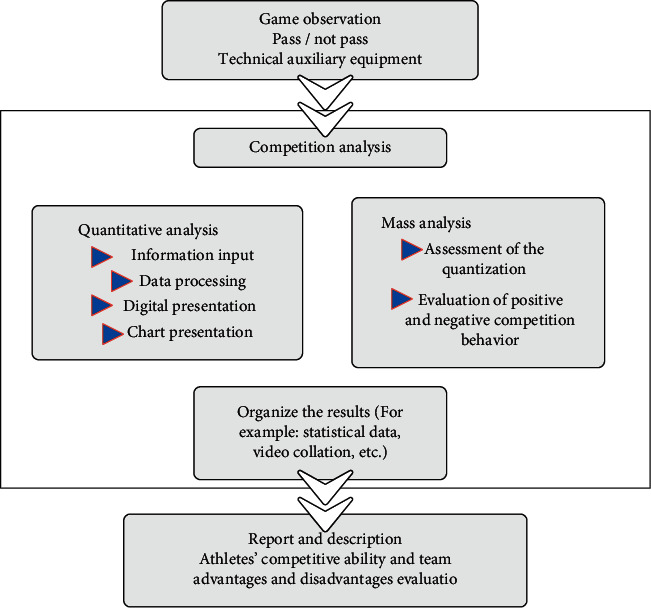
Schematic diagram of the game observation steps of the system.

The competition is observed and analyzed to gain important information related to the science of training. This information pertains to psychological and physical characteristics that limit athletic ability, technical and tactical abilities, team leadership, and the development of formations in team games such as soccer.  Figure 9 shows a schematic diagram of the game observation steps of the system.

Based on the above research, the effect of the e-sports competition analysis system proposed in this paper is verified, the data analysis effect of e-sports competition is counted, its prediction effect is studied, and the results shown in Tables 1 and 2 are obtained.

From the above research, it can be seen that the e-sports competition analysis system based on the intelligent analysis system proposed in this paper has a good e-sports competition analysis effect, and has a good effect in e-sports competition prediction.

## 4. Conclusion

As a newly developed sport, e-sports integrates culture, technology, intelligence, and sports. Healthy e-sports is fundamentally different from online games. In essence, it regards electronic equipment and electronic technology as sports equipment to compete against each other in the specified time according to the unified competition standard in the virtual information technology environment it has set up. Moreover, e-sports is not only an intellectual exercise, but also a form of exercise that integrates the mind and body. It can not only promote the improvement of participants' responsiveness, thinking ability, teamwork, and stress resistance, but also better realize the ideals and values of participants. The research results show that the e-sports competition analysis system based on the intelligent analysis system proposed in this paper has a good e-sports competition analysis effect, and has a good effect in e-sports competition prediction.

## Figures and Tables

**Table 1:** The analysis effect of e-sports competition analysis system based on intelligent analysis system on e-sports competition data.

Number	Analysis of e-sports competitions
1	80.39
2	75.32
3	76.35
4	77.87
5	83.03
6	85.44
7	75.29
8	85.99
9	73.83
10	85.22
11	83.86
12	76.69
13	84.50
14	82.10
15	71.14
16	84.51
17	78.00
18	78.48
19	81.48
20	80.96
21	74.59
22	84.10
23	80.16
24	72.61
25	74.18
26	73.92
27	75.06
28	74.81
29	77.60
30	86.58

**Table 2:** The prediction effect of the e-sports competition analysis system based on the intelligent analysis system on the e-sports competition.

Number	E-sports predictions
1	63.56
2	62.63
3	54.29
4	55.14
5	54.28
6	57.00
7	53.98
8	58.10
9	56.37
10	57.32
11	57.25
12	60.09
13	57.57
14	59.27
15	55.50
16	54.67
17	54.24
18	54.12
19	63.88
20	57.17
21	58.92
22	63.12
23	57.80
24	56.32
25	60.52
26	53.46
27	54.41
28	54.39
29	53.34
30	54.06

## Data Availability

The labeled data set used to support the findings of this study are available from the corresponding author upon request.
